# Enhancing very long chain fatty acids production in *Yarrowia lipolytica*

**DOI:** 10.1186/s12934-022-01866-6

**Published:** 2022-07-11

**Authors:** Peter Gajdoš, Veronika Urbaníková, Mária Vicenová, Milan Čertík

**Affiliations:** Institute of Biotechnology, Faculty of Chemical and Food Technology, Slovak University of Technology, Radlinského 9, 81237 Bratislava, Slovak Republic

**Keywords:** *Yarrowia lipolytica*, Erucic acid, Behenic acid, Diacylglycerol acyltransferase

## Abstract

**Background:**

Very long chain fatty acids (VLCFA) and their derivatives are industrially attractive compounds. The most important are behenic acid (C22:0) and erucic acid (C22:1Δ^13^), which are used as lubricants, and moisturizers. C22:0 and C22:1Δ^13^ have also potential for biofuel production. These fatty acids are conventionally obtained from plant oils. *Yarrowia lipolytica* is an oleaginous yeast with a long history of gene manipulations resulting in the production of industrially interesting compounds, such as organic acids, proteins, and various lipophilic molecules. It has been shown previously that it has potential for the production of VLCFA enriched single cell oils.

**Results:**

The metabolism of *Y. lipolytica* was redesigned to achieve increased production of VLCFA. The effect of native diacylglycerol acyltransferases of this yeast YlLro1p, YlDga1p, and YlDga2p on the accumulation of VLCFA was examined. It was found that YlDga1p is the only enzyme with a beneficial effect. Further improvement of accumulation was achieved by overexpression of 3-ketoacyl-CoA synthase (*TaFAE1*) under *8UAS-pTEF* promoter and blockage fatty acid degradation pathway by deletion of *YlMFE1*. The best-producing strain YL53 (*Δmfe, pTEF-YlDGA1*, *8UAS-pTEF-TaFAE1*) produced 120 µg of very long chain fatty acids per g of produced biomass, which accounted for 34% of total fatty acids in biomass.

**Conclusions:**

Recombinant strains of *Y. lipolytica* have proved to be good producers of VLCFA. Redesign of lipid metabolism pathways had a positive effect on the accumulation of C22:1Δ^13^ and C22:0, which are technologically attractive compounds.

## Background

Very long chain fatty acids (VLCFA) are fatty acids longer than 20 carbons synthesized by the complex of fatty acid elongase. Fatty acid elongases are structurally and functionally very similar to fatty acid synthases. Both possess four enzymes performing a sequence of condensation, reduction, dehydratation, and reduction reactions. The major difference is that fatty acid synthases use acetyl-CoA as a starting substrate while elongases use exclusively fatty acyl-CoA instead. In plants substrate specificity of elongases is conferred by 3-ketoacyl-CoA synthase, which is the first enzyme in the four-step sequence, and it is performing condensation reaction. Plant genes responsible for 3-ketoacyl-CoA synthase activity are *FATTY ACID ELONGATION* (*FAE1*) [1]. Interestingly, FAE1-ketoacyl-CoA synthases are structurally different from yeast Elo proteins [2]. Compared to yeasts, which accumulate VLCFA only in minor quantities, plants are very good producers of VLCFA [1, 3]. Since VLCFA are components of cuticular waxes, they are essential for all plants [4]. Especially plants from the Brassicaceae family are exceptional producers of VLCFA, which are accumulated in seed oils of these plants in the form of triacylglycerols [5]. In seed oils of *Simmondsia chinensis*, VLCFA are stored in the form of waxes (esters of fatty acids and fatty alcohols) [6].

VLCFA and their derivatives have many industrial applications. For example, erucic acid (C22:1Δ^13^) is applicable as a surfactant, lubricant, and constituent of nanocomposites [7, 8]. A study by Seames and colleagues has described the beneficial impact of C22:1Δ^13^ on biodiesel yield produced by non-catalytic cracking of triacylglycerols [9]. Xu and colleagues described the preparation of diesel-like hydrocarbons from behenic acid (C22:0) [10]. C22:0 and its derivative behenyl alcohol are common constituents of cosmetics as moisturizers [11]. Derivatives of C22:1Δ^13^ and C22:0, erucamide and behenamide, are used as slip agents [12].

Heterologous expression of plant FAE1-ketoacyl-CoA synthases in yeasts led to the production of saturated and monounsaturated VLCFA. In the study by Fillet and colleagues, *Rhodosporidium toruloides* was used as a host for heterologous expression of four *FAE1* from *Arabidopsis thaliana, Cardamine graeca, Crambe abyssinica, and Lunaria annua* [13]. VLCFA profile and overall percentage of VLCFA were influenced by specific *FAE1*. Similarly, *FAE1* from *Thlaspi arvense* (*TaFAE1*) was successfully expressed in *Yarrowia lipolytica*, which was cultivated on cheap feedstocks [14]. Yeasts have certain advantages over plants, especially much shorter generation periods and independence from climate and seasons. However, heterologous production of desired metabolites in yeasts often requires several steps of genetic manipulations. It was shown previously, that besides genes directly responsible for fatty acid synthesis, genes regulating storage lipid metabolism such as acyltransferases are very important for the accumulation of specific fatty acids [15, 16].

*Yarrowia lipolytica* is ascomycetous, non-pathogenic, oleaginous yeast. Due to its natural ability to produce extracellular proteins, organic acids, and intracellular lipids, it was predestined to become a model yeast for studies concerning the production of such compounds [17, 18]. Moreover, there is a plethora of genetic engineering tools developed for manipulating the genome of *Y. lipolytica* [19]. Thanks to these features it has become the cellular factory for the production of many biotechnologically interesting compounds [20]. As mentioned above, VLCFA were already produced in *Y. lipolytica* in moderate quantities. This study was performed to elucidate if it is possible to redesign the lipid metabolism of *Y. lipolytica* to further increase the production of VLCFA. Three approaches were assessed if they could work in synergy to obtain increased VLCFA production. Initially, the impact of native diacylglycerol acyltransferases from *Y. lipolytica* on VLCFA accumulation was studied. Subsequently, the effect of *TaFAE1* controlled by strong promoter *8UAS-pTEF* on VLCFA production was examined. Finally, the influence of *Δmfe* genotype on VLCFA production was evaluated. The combination of these three strategies proved beneficial for VLCFA production.

## Results

### **Co-expression of*FAE1*with different diacylglycerol acyltransferase genes**

**Fig. 1:**
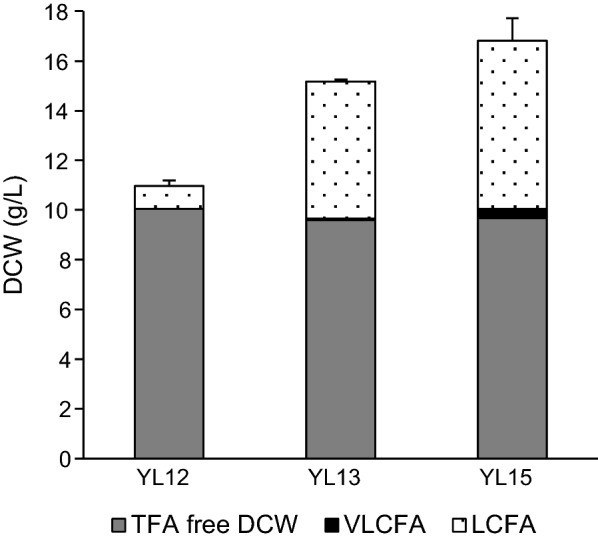
Biomass production of YL12 (*pTEF-YlLRO1*, *pTEF-TaFAE1*), YL13 (*pTEF-YlDGA2*, *pTEF-TaFAE1*), and YL15 (*pTEF-YlDGA1*, *pTEF-TaFAE1*). YL12, YL13, and YL15 were grown in a 60 g/L glucose medium with a C/N ratio of 80 for 72 h. Each value is an average of three experiments. DCW-dry cell weight, TFA-total fatty acids

**Fig. 2:**
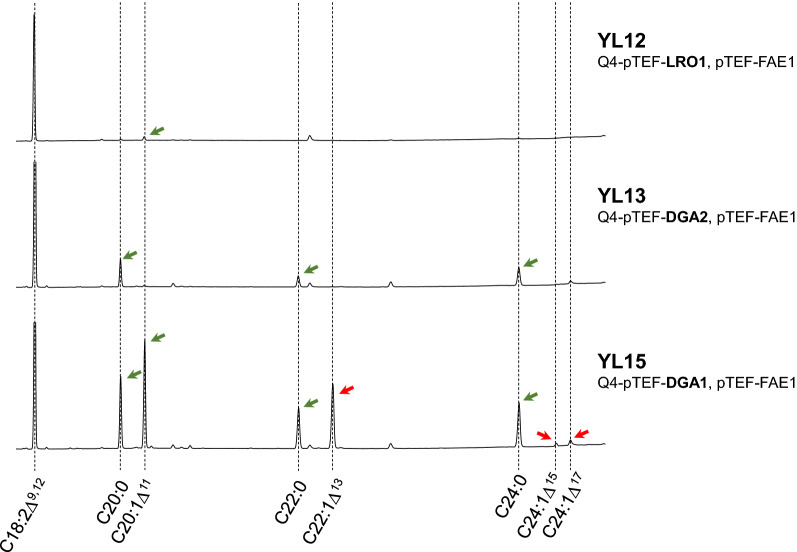
Very long chain fatty acids in strains YL12 (*pTEF-YlLRO1*, *pTEF-TaFAE1*), YL13 (*pTEF-YlDGA2*, *pTEF-TaFAE1*), and YL15 (*pTEF-YlDGA1*, *pTEF-TaFAE1*). Green arrows indicate fatty acids natural for *Y. lipolytica* increased by expression of *TaFAE1.* Red arrows indicate fatty acids synthesized in recombinant strains expressing *TaFAE1* which are not found in wild type of *Y. lipolytica* W29. Increased production of very long chain fatty acids was observed only in the strain YL15

The 3-ketoacyl-CoA synthase encoded by *TaFAE1* from *Thlaspi arvense* was expressed in recombinant strains of *Y. lipolytica.* Strains JMY1882 (*pTEF-YlLRO1*), JMY1884 (*pTEF-YlDGA2*) and JMY1892 (*pTEF-YlDGA1*) [21] were used as a host for *TaFAE1* resulting in strains YL12 (*pTEF-YlLRO1*, *pTEF-TaFAE1*), YL13 (*pTEF-YlDGA2*, *pTEF-TaFAE1*), and YL15 (*pTEF-YlDGA1*, *pTEF-TaFAE1*). YL12, YL13, and YL15 have shown different growth patterns after 72 h of cultivation in a medium with 60 g/L glucose as a carbon substrate (Fig. 1). Higher biomass yields in YL13 and YL15 were accompanied by a significant difference (p-value < 0.05) in lipid accumulation. While the strain YL12 produced less than 10% of total fatty acids (TFA) in biomass, both YL13 and YL15 accumulated more than 30% of TFA (YL13–37%, YL15–43%). Expression of *TaFAE1* resulted in the effective production of VLCFA only in the strain YL15 (Fig. 2). The other two strains exhibit neither increase in saturated nor in monounsaturated VLCFA.

**Fig. 3:**
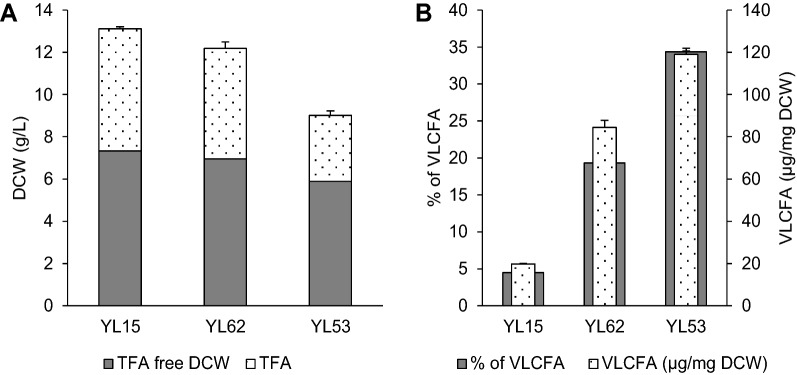
Biomass production (**A**) and very long chain fatty acid accumulation (**B**) in yeast strains YL15 (*pTEF-YlDGA1*, *pTEF-TaFAE1*), YL62 (*pTEF-YlDGA1*, *8UAS-pTEF-TaFAE1*), and YL53 (*Δmfe, pTEF-YlDGA1*, *8UAS-pTEF-TaFAE1*). All strains were grown in a 60 g/L glucose medium with a C/N ratio of 80 for 72 h. Each value is an average of three experiments. DCW-dry cell weight, TFA-total fatty acids, VLCFA-very long chain fatty acids

## Improvement of VLCFA accumulation by redesign of metabolism

Since the accumulation of VLCFA by the strain YL15 was quite low (approximately 4.5% of TFA) strategy for increasing VLCFA content was conducted. First, instead of the *pTEF* promoter stronger *8UAS-pTEF* promoter was used to control the expression of *TaFAE1.* This change resulted in a more than 4-fold increase of VLCFA percentage in TFA of resulting strain YL62 (*pTEF-YlDGA1*, *8UAS-pTEF-TaFAE1*) compared to strain YL15. Another improvement was achieved by the abolition of β-oxidation. VLCFA percentage in the strain YL53 (*Δmfe, pTEF-YlDGA1*, *8UAS-pTEF-TaFAE1*) was increased more than 7-fold compared to YL15. Strains YL15, YL62, and YL53 were grown for 72 h in a medium with 60 g/L glucose which was still present after the end of the cultivation (Table 1). Accumulation of TFA in biomass in strains YL15, YL62, and YL53 was 44%, 43%, and 35%, respectively.

**Table 1:** Residual glucose (Glc), mannitol (Man), and citric acid (CA) in medium

	Glc (g/L)	Man (g/L)	CA (g/L)
YL15	17.4 ± 0.6	2.1 ± 0.1	4.4 ± 0.1
YL62	15.0 ± 0.8	2.4 ± 0.5	3.2 ± 0.5
YL53	19.5 ± 0.1	0.8 ± 0.1	2.1 ± 0.1

Yeast strains YL15 (*pTEF-YlDGA1*, *pTEF-TaFAE1*), YL62 (*pTEF-YlDGA1*, *8UAS-pTEF-TaFAE1*), and YL53 (*Δmfe, pTEF-YlDGA1*, *8UAS-pTEF-TaFAE1*) were grown in 60 g/L glucose medium with C/N ratio of 80 for 72 h. Each value is an average of three experiments

It was observed, that increasing VLCFA accumulation was accompanied by a drop in biomass yield (Fig. 3). Nonetheless, strain YL53 was the best VLCFA producer yielding up to 120 µg of VLCFA per g of produced biomass, which accounted for 34% of TFA in biomass.

## Fatty acid profiles of VLCFA-producing strains

**Fig. 4:**
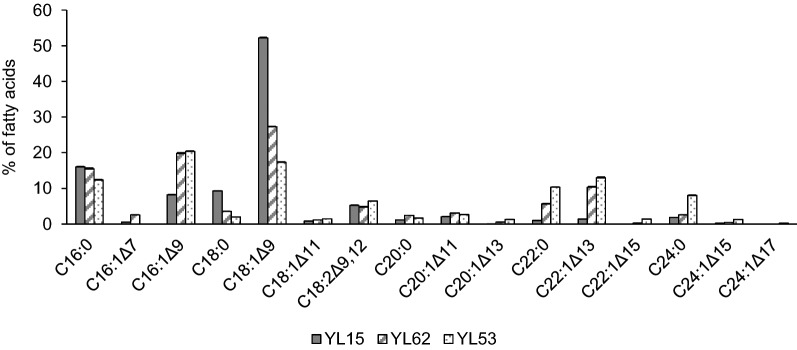
Fatty acid profiles of strains YL15 (*pTEF-YlDGA1*, *pTEF-TaFAE1*), YL62 (*pTEF-YlDGA1*, *8UAS-pTEF-TaFAE1*), and YL53 (*Δmfe, pTEF-YlDGA1*, *8UAS-pTEF-TaFAE1*). All strains were grown in a 60 g/L glucose medium with a C/N ratio of 80 for 72 h. Each value is an average of three experiments

Fatty acid profiles of constructed strain differed according to changes in the genome of cells (Fig. 4). The fatty acid profile of YL15 was very close to the standard fatty acid profile of wild type strain W29 with oleic acid (C18:1Δ^9^) as the major fatty acid [18]. The major difference was the production of C22:1Δ^13^ which is not common fatty acid in *Y. lipolytica.* The situation was changed by overexpression of *TaFAE1* under the control of *8UAS-pTEF* in YL62 when C18:1Δ^9^ dropped from over 50% to less than 30% of TFA in cells. This was accompanied by a more than 2-fold increase in palmitoleic acid (C16:1Δ^9^) and an important increase in all VLCFA. The most profound change in fatty acid profile was seen in strain YL53, where the major fatty acid became C16:1Δ^9^ instead of the usual C18:1Δ^9^. Behenic (C22:0), C22:1Δ^13^, and lignoceric (C24:0) acids were the most accumulated VLCFA in YL53. C22:1Δ^13^ was the most abundant VLCFA representing 13% of TFA.

## Discussion

In the previous study, it was shown that expression of *TaFAE1* from *Thlaspi arvense* in *Y. lipolytica* induced monounsaturated VLCFA production in this yeast [14]. Since the production of VLCFA in recombinant *Y. lipolytica* was quite low compared to plants, further manipulation was required to increase VLCFA content in this yeast. Generally, the accumulation of fatty acids is closely connected to the ability of the cell to sequester it to specific subcellular compartments such as lipid droplets. Fatty acids in lipid droplets are usually stored in the form of triacylglycerols, which are synthesized by diacylglycerol acyltransferases. Diacylglycerol acyltransferases are divided into phospholipid:diacylglycerol acyltransferases (PDAT) and acyl-CoA:diacylglycerol acyltransferases (DGAT) according to the acyl donor. DGAT are further categorized into DGAT1 and DGAT2 families according to their structure. In *Y. lipolytica* three genes are coding diacylglycerol acyltransferase: *YlLRO1*, *YlDGA1*, and *YlDGA2*. Previously, strains carrying a single diacylglycerol acyltransferase enzyme were constructed [21]. In this study, these strains were used as a host for *TaFAE1.* It was observed that only strain co-expressing *TaFAE1* and *YlDGA1* (YL15) has shown considerable VLCFA accumulation. The other two strains (YL12 and YL13) exhibited neither increase in C22:0 and C24:0 which are naturally synthesized also in wild type strain W29 [22] nor in C22:1Δ^13^ and C24:1Δ^15^ fatty acids, which are synthesized exclusively by the action of TaFae1p. These results indicate that only YlDga1p has the preference for a very long chain fatty acyl-CoA. YlLro1p and YlDga2p did not provide accumulation of VLCFA. YlLro1p has the role of PDAT in *Y. lipolytica* and it has only a minor contribution to triacylglycerol accumulation. Its main role is rather phospholipid remodelling through its phospholipase activity. On the other hand, *YlDGA1* coding a DGAT2 acyltransferase and *YlDGA2* coding a DGAT1 acyltransferase has a major impact on triacylglycerol accumulation in *Y. lipolytica*. While it is common for plants and animals to harbour both DGAT1 and DGAT2 enzymes, *Y. lipolytica* and *Blastobotrys raffinosifermentans* are the only two known microorganisms with both enzymes [21, 23]. YlDga1p and YlDga2p have different localisation in cells of *Y. lipolytica* [24] and obviously have different substrate specificities. Preference of DGAT1 and DGAT2 enzymes for certain types of acyl-CoA is not unusual and it was described for example in *Vernicia fordii* [25] and *Claviceps purpurea* [26]. More recently, the results of Sanya and colleagues indicate, that Dga1p and Dga2p from *B. raffinosifermentans* may have different specificities for substrates [23]. Moreover, the preference of certain enzymes for fatty acids of specific lengths is not uncommon in *Y. lipolytica*. Specifically, enzymes of fatty acid transport and activation machinery have different substrate specificities towards fatty acids of different chain lengths. Long chain fatty acids are preferentially activated by YlFaa1p in the cytosol, while fatty acids with shorter chains are directly transported to peroxisomes [27].

Further improvement of VLCFA production was achieved by tuning the *TaFAE1* expression. The simple replacement of the *pTEF* promoter for *8UAS-pTEF*, which is stronger than *pTEF* [28], increased the VLCFA content from approximately 20 to more than 80 µg/mg of biomass in strains YL15 and YL62, respectively. This confirmed the premise, that increase in promoter strength results in higher enzymatic activity and ultimately in elevated VLCFA production. One of the possible explanations why the higher activity of TaFae1p could improve VLCFA production is connected to the availability of acyl-CoA used as precursors for VLCFA synthesis. Generally, stearoyl-CoA (C18:0-CoA) and oleoyl-CoA (C18:1Δ^9^-CoA) are molecules utilized by elongases for VLCFA production [29]. However, these molecules are utilized also by desaturases and acyltransferases. In *Y. lipolytica* two fatty acid desaturases are present: YlOle1p and YlFad2p. Generally, Ole1p works as Δ9-desaturase and its activity is crucial for cell viability [30]. Ole1p desaturates palmitoyl-CoA (C16:0-CoA) and C18:0-CoA to their monounsaturated forms. This is beneficial for C22:1Δ^13^ synthesis by elongase since C18:1Δ^9^-CoA is its precursor. Fad2p introduces a second double bond on the 12th carbon of C18:1Δ^9^ from the carboxyl end. At first glance, Fad2p looks like the most direct competitor of elongase for the substrate. However, in a study by Li and colleagues, it is visible, that silencing of *CaFAD2* in *Crambe abyssinica* had a lesser impact on C22:1Δ^13^ production than improvement of elongation and C22:1Δ^13^ incorporation into triacylglycerols [31]. Similar results were obtained in our laboratory when expression of *TaFAE1* in *Δfad2* mutant of *Y. lipolytica* resulted only in 2% higher production of C22:1Δ^13^ than in the control strain expressing *TaFAE1* (unpublished data). It could be concluded, that the *fad2* genotype is beneficial but provides only a small improvement in VLCFA production. On the other hand, enhancement of elongation activity proved as more important for increased VLCFA production than the loss of Δ12-desaturase activity in plants and yeasts, as well [13, 31]. Our results suggest that overexpression of *TaFAE1* provided higher elongase activity, which prevented 18 carbon long acyl-CoA from being captured by acyltransferases and incorporated into either phospholipids or triacylglycerols. These acyl-CoAs were processed by elongases first and then became available for acyltransferases. An analogous situation was observed when competition between glycerol-3-phosphate acyltransferase Sct1p and Δ9-desaturase Ole1p was studied in *Saccharomyces cerevisiae.* Overexpression of *SCT1* decreased the desaturation of fatty acids while overexpression of *OLE1* increased the desaturation of fatty acids in this yeast [32].

Since VLCFA as well as other fatty acids could undergo degradation in the β-oxidation pathway, it is desirable to manipulate this catabolic pathway. Generally, there are two approaches described in the literature on how to prevent unwanted fatty acid degradation by β-oxidation. The first is to remove acyl-CoA oxidase activity, which is in *Y. lipolytica* represented by six *POX1-6* [33]. The second is to delete *MFE1*, the gene which is encoding the multifunctional enzyme responsible for all β-oxidation reactions except acyl-CoA oxidation [34]. In this study strain carrying a deletion of *YlMFE1* was employed to test if the blockage of the β-oxidation pathway could improve VLCFA accumulation. It must be said that deletion of *YlMFE1* had a negative impact on biomass yield, which was observed previously as well [22]. Moreover, strain YL53 accumulated 35% of TFA in biomass which was about 10% less than two other strains YL15 and YL62. Nonetheless, YL53 was the best producing strain yielding 120 µg of VLCFA per mg of biomass. The total lipid content in our strains is comparable to data published by other research groups. Many studies using either wild type or recombinant strains designed for lipid accumulation have described lipid contents as about 25–50% of biomass [35–39]. Exceptional lipid production of over 92% of biomass was achieved by Ledesma-Amaro and colleagues [40]. However, such very high production of lipids was achieved due to fatty acid secretion phenotype.

The overall percentage of VLCFA in our best strain YL53 was 34%, which is comparable to strains constructed by Fillet and colleagues, where total VLCFA ranged from 30 to 40% of TFA. Profile of VLCFA differed according to the source of *FAE1* since sequences were obtained from *A. thaliana, C. graeca, C. abyssinica, and L. annua.* They have found that 3-ketoacyl-CoA synthase from *C. graeca* was better for nervonic acid (C24:1Δ^15^) production, while 3-ketoacyl-CoA synthase from *C. abyssinica* increased C22:1Δ^13^ content [13]. In our strains the most produced VLCFA was C22:1Δ^13^. This is in line with VLCFA production in *T. arvense*, where C22:1Δ^13^ represents almost 40% of TFA, while other VLCFA are produced only in minor quantities [5]. In our best-producing strain *Y. lipolytica* YL53 also C22:0 and C24:0 are quite abundant in addition to C22:1Δ^13^.

## Conclusions

In this study, *Y. lipolytica* was manipulated with the aim of enhanced VLCFA production. *YlLRO1*, *YlDGA1*, and *YlDGA2* were co-expressed with *TaFAE1* to determine the most suitable native diacylglycerol acyltransferase from *Y. lipolytica* for VLCFA production. We have found, that YlDga1p acyltransferase has a positive impact on VLCFA accumulation. Further improvement in VLCFA accumulation was achieved by the expression of *TaFAE1* using a strong *8UAS-pTEF* promoter. Deletion of *YlMFE1* had a negative impact on the biomass yield, nonetheless further improved VLCFA accumulation. Thus, strain producing more than 30% of VLCFA was constructed with C22:1Δ^13^ as the most produced followed by C22:0 and C24:0. C22:1Δ^13^ and C22:0 are technologically interesting compounds and microbial production of oils enriched with these fatty acids is an interesting alternative to their production by plants.

## Methods

### Strains, media composition, and culture conditions

*Yarrowia lipolytica* and *Escherichia coli* strains used in this study are summarized in Table 2.

**Table 2:** *Escherichia coli* and *Y. lipolytica* strains and plasmids used in this study

Strain (host strain)	Plasmid, genotype	References
*E. coli* strains		
EC11 (DH5a)	JMP62*-pTEF-TaFAE1-LEU2ex*	[14]
EC61 (DH5a)	JMP62*-8UAS-pTEF-TaFAE1-LEU2ex*	This work
JME1112 (DH5a)	JMP62*-pTEF-YlDGA1-URA3ex*	[21]
JME4305 (DH5a)	JMP62*-URA3ex-8UAS-pTEF-FAR4*	[41]
*Y. lipolytica* strains		
JMY1882	*MATA ura3-302 leu2-270 xpr2-322 Δdga1Δlro1Δare1Δdga2 pTEF-YlLRO1-URA3ex*	[21]
JMY1884	*MATA ura3-302 leu2-270 xpr2-322 Δdga1Δlro1Δare1Δdga2 pTEF-YlDGA2-URA3ex*	[21]
JMY1892	*MATA ura3-302 leu2-270 xpr2-322 Δdga1Δlro1Δare1Δdga2 pTEF-YlDGA1-URA3ex*	[21]
JMY1915	*MATA ura3-302 leu2-270 xpr2-322 Δdga1Δlro1Δare1Δdga2Δmfe*	[34]
YL12	JMY1882 *pTEF-TaFAE1-LEU2ex*	This work
YL13	JMY1884 *pTEF-TaFAE1-LEU2ex*	This work
YL15	JMY1892 *pTEF-TaFAE1-LEU2ex*	This work
YL51	JMY1915 *pTEF-YlDGA1-URA3ex*	This work
YL53	JMY1915 *pTEF-YlDGA1-URA3ex 8UAS-pTEF-TaFAE1-LEU2ex*	This work
YL62	JMY1892 *8UAS-pTEF-TaFAE1-LEU2ex*	This work

*Escherichia coli* were cultured in a lysogeny broth medium supplemented with 50 µg/mL kanamycin, according to a standard protocol [42]. All *Y. lipolytica* strains used in this study are derived from strain W29 (ATCC 20,460). The plates of minimal YNB and YNBleu media agar were used for the selection of transformants. The minimal YNB medium consisted of 0.17% (w/v) yeast nitrogen base (without amino acids and ammonium sulfate; BD, Erembodegem, Belgium), 0.5% (w/v) NH_4_Cl, 50 mM phosphate buffer (pH 6.8), and 2% (w/v) glucose. For the YNBleu medium, leucine (0.1 g/L) was added to the YNB medium. Agar plates were prepared by the addition of 20 g/L agar. Yeast inoculum was prepared in rich YPD medium containing 1% (w/v) yeast extract (BD, Erembodegem, Belgium), 1% (w/v) peptone (BD, Erembodegem, Belgium), and 2% (w/v) glucose (Mikrochem, Pezinok, Slovakia). The medium for lipid production (C/N ratio of 80) contained 60 g/L glucose, 1.5 g/L yeast extract, 0.5 g/L NH_4_Cl, 7 g/L KH_2_PO_4_, 5 g/L Na_2_HPO_4_.12H_2_O, 0.1 g/L CaCl_2_, 1.5 g/L MgSO_4_.7H_2_O, 10 mg/L ZnSO_4_.7H_2_O, 0.6 mg/L FeCl_3_.6H_2_O, 0.07 mg/L MnSO4.H_2_O, and 0.04 mg/L CuSO_4_.5H_2_O. The medium for lipid production was filter sterilized. Yeast inoculum was prepared in 20 mL of YPD medium in 100 mL flasks. Subsequently, 50 mL of production medium in 250 mL baffled flasks were inoculated with a 24-hour inoculum having an optical density (OD600) of 0.1. The cells were cultured at 28 °C on an orbital shaker at 130 rpm. Experiments were carried out in three biological replicates.

### Plasmid and strain construction

*TaFAE1* from *Thlaspi arvense* (GenBank Accession Number KT223025.1) was codon-optimized for *Y. lipolytica* as described previously [14]. *E. coli* strain EC61 (JMP62*-8UAS-pTEF-TaFAE1-LEU2ex*) was constructed in this work. Plasmids (JMP62*-pTEF-TaFAE1-LEU2ex*) from the *E. coli* EC11 [14] and (JMP62*-URA3ex-8UASpTEF-FAR4*) from *E. coli* JME4305 [41] were double-digested by ClaI and BamHI to replace *pTEF* promoter in (JMP62*-pTEF-TaFAE1-LEU2ex*) by *8UAS-pTEF* from (JMP62*-URA3ex-8UAS-pTEF-FAR4*). The resulting plasmid (JMP62-*8UAS-pTEF-TaFAE1-LEU2ex*) was stored in *E. coli* EC61. Insertion cassettes obtained by the digestion of plasmids from EC61 and JME1112 (JMP62*-pTEF-YlDGA1-URA3ex*) [21] using NotI (New England Biolabs, Ipswich, MA, USA) were used for the transformation of yeast cells. Roti^®^-Prep Plasmid MINI and Roti^®^-Prep Gel Extraction kits (Carl Roth, Karlsruhe, Germany) were used for plasmid extraction and recovery of DNA fragments from agarose gel, respectively. The transformation of the yeast cells was done by the lithium acetate method [43]. The genomic DNA was prepared according to Lõoke and colleagues [44] and was then amplified by PCR in a Bio-Rad T100™ Thermal cycler using GoTaq® DNA polymerase (Promega, Madison, WI, USA). The successful insertion of *DGA1* was verified using the primer pair URA3in and DGA1in with sequence 5′- TTGGTGGTGGTAACATCCAGAG-3′ and 5′- AGCCAGATGATTCTCCACGG-3′, respectively. The successful insertion of *TaFAE1* was verified using the primer pair LEU2in and FAE1in with sequence 5′- TACGACGCATTGATGGAAGG-3′ and 5′- TTCACCACCATAGCGGACAG-3′, respectively.

### Analytical methods

Isolation of biomass was performed as follows. Cell suspensions were centrifuged (2880 ×*g*, 5 min), washed twice with saline, once with deionized water, and freeze-dried. The freeze-dried cells were used for lipid analysis. The dry cell weight (DCW) was determined gravimetrically. Residual glucose, citric acid, and mannitol were measured by HPLC (Agilent Technologies, Santa Clara, CA, USA) using an Aminex HPX87H column (Bio-Rad, Hercules, CA, USA) coupled to an RI detector and UV detector, as described previously [45]. Fatty acids from freeze-dried cells were transformed into FA methyl esters and analysed as described previously [22].

### Data analysis

Statistical analysis was performed by the software Microsoft Excel (Microsoft Office 365 software pack) equipped with the Data analysis tool. Obtained data were processed with the Single Factor Analysis of Variance (ANOVA).

## Data Availability

All data generated or analysed during this study are included in this published article.
